# NO-H_2_S interactions involve cGMP

**DOI:** 10.1186/2050-6511-14-S1-O32

**Published:** 2013-08-29

**Authors:** Andreas Papapetropoulos

**Affiliations:** 1 Laboratory for Molecular Pharmacology, Department of Pharmacy, University of Patras, Greece

## Background

Hydrogen sulfide (H_2_S) and nitric oxide (NO) have been recognized as endogenous signaling molecules, involved in a variety of homeostatic and disease processes. Although it is well-established that NO increases cGMP content of cells and tissues by activating soluble guanylyl cyclase (sGC), the ability of H_2_S to affect cyclic nucleotides levels has been controversial.

## Results

We have shown that H_2_S increases cGMP in both endothelial and smooth muscle cells. However, H_2_S does not activate sGC or alter NO-induced sGC activity. Interestingly, H_2_S inhibits phosphodiesterase (PDE) activity; although it reduces PDE activity of several PDE H_2_S is most effective and potent against PDE-5. IN line with the ability of H_2_S to increase cellular cGMP, we observed that exposure of cells to H_2_S leads to activation of cGMP-dependent protein kinase and VASP phosphorylation. As both NO and H_2_S promote angiogenesis and vasodilation we explored their interactions in the vessel wall in the context of these two biological processes. Inhibition of eNOS or PKG reduced the H_2_S-stumlated angiogenic properties of endothelial cells, as well as H_2_S–stimulated vasorelaxation, suggesting a prominent role for cGMP/PKG pathways in H_2_S signaling. On the other hand, silencing of the H_2_S-producing enzyme cystathionine-γ-lyase (CSE) reduced NO-stimulated cGMP accumulation, angiogenesis and smooth muscle relaxation, proving that NO requires H_2_S to manifest its effects. Finally, H_2_S-induced wound healing and angiogenesis in vivo was suppressed by pharmacological inhibition or genetic ablation of eNOS.

## Conclusion

Inhibition of the production of one gasotransmitter (NO or H_2_S) reduces the ability of the other to elevate cGMP and to trigger angiogenesis and vasodilation. These observations establish the existence of a positive, synergistic cross-talk between H_2_S and NO in vascular tissues.

